# Clinical outcomes of total endovascular aortic arch repair by in situ microneedle fenestration using the triple (stiff)-sheathed gutter technique

**DOI:** 10.1186/s42155-026-00788-3

**Published:** 2026-10-29

**Authors:** Peng Ye, Hongfei Miao, Qingle Zeng, Wenya Liu, Mingyuan Ma, Yong Chen

**Affiliations:** 1 Division of Vascular and Interventional Radiology, Department of General Surgery, Nanfang Hospital, Southern Medical University, No. 1838, North of Guangzhou Avenue, Guangzhou City, Guangdong Province China; 2 Department of Vascular Intervention, Shantou Central Hospital, Shantou, China

**Keywords:** Aortic arch, Aortic arch pathology, Total endovascular aortic arch repair, In situ fenestration, Regional cerebral oxygen saturation

## Abstract

**Purpose:**

To investigate the outcomes of total endovascular aortic arch repair (TEAAR) via in situ micro-needle fenestration (ISMNF) with the triple (stiff)-sheathed gutter technique.

**Materials and methods:**

In this retrospective study, the data of consecutive patients who underwent TEAAR via ISMNF with triple (stiff)-sheathed gutter technique between August 2022 and April 2025 were analyzed. Three 6-Fr, 90-cm sheaths were placed into ascending aorta without removing the dilator and super-stiff guidewires during main stent-graft deployment. This configuration creates a gutter between the stent-graft and aortic wall to maintain perfusion to supra-aortic branches. Operative details, technical success, 30-day and midterm outcomes, and adverse events were recorded. Kaplan–Meier analysis was used to estimate freedom from all-cause death and reintervention.

**Results:**

Overall, 36 patients were included. The technical success rate was 94.4% (34/36). Endovascular reconstruction of cervical branches was successfully performed in all cases. The 30-day mortality was 2.8%. The median follow-up period was 19 months (range, 1–32 months). The all-cause death rate was 11.1% (4/36), with no cases of any aorta-related death. The prevalence of endoleaks during follow-up was 13.9% (5/36). The overall stroke rate was 8.3% (3/36). The 1- and 2-year survival rates were 97.3% ± 2.7% and 85.8% ± 8.1%, respectively. The cumulative rate of freedom from reintervention at 1 and 2 years was 87.5% ± 5.9% and 83.2% ± 7.0%, respectively.

**Conclusion:**

TEAAR via ISMNF with the triple (stiff)-sheathed gutter technique was performed with a 94% technical success rate and stroke risk similar to open surgery.

## Introduction

Thoracic endovascular aortic repair (TEVAR) is a widely used treatment for thoracic aortic aneurysm and complicated type B aortic dissection. However, total arch replacement is still the primary first-line treatment requiring median sternotomy under hypothermic circulatory arrest with significant mortality and morbidity [1–3]. Several commercial devices for aortic arch repair have emerged in recent years; most still require debranching bypass, surgical arteriotomy, or cardiopulmonary bypass.

Endovascular techniques are less invasive and enable treatment of high-risk patients. Design improvements in endovascular repair devices for aortic arch pathologies and advances in TEVAR techniques for cervical branch endovascular reconstruction have resulted in successful total endovascular aortic arch repair (TEAAR) in zone 0 [4–13]. Successful TEAAR by in situ needle fenestration has been reported [4–6, 8, 9].

In the present study, ISMNF was used to revascularize all cervical branches during TEAAR using the triple (stiff)-sheathed gutter technique to maintain cervical perfusion. Herein, the early and midterm clinical outcomes of this technique are described.

## Methods

### Study design and inclusion/exclusion criteria

The study protocol for this retrospective study was approved by the institutional review board. All patients provided written informed consent for the use of anonymized clinical data for research purposes before treatment.

**Fig. 1:**
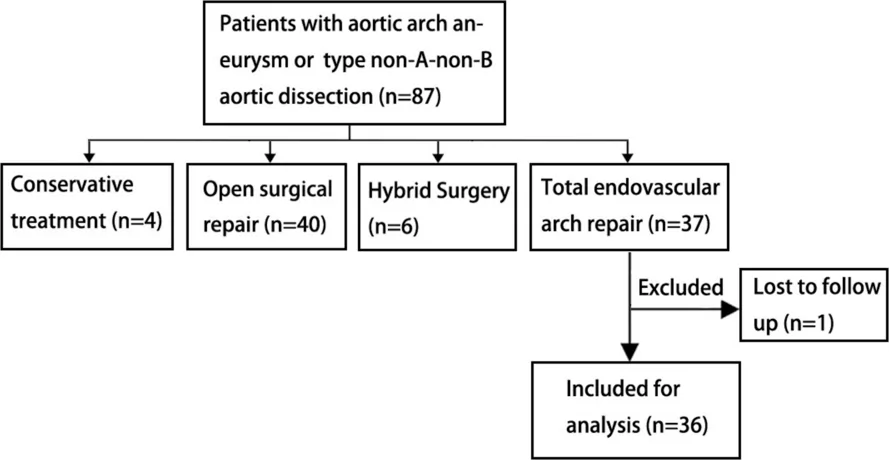
Patient inclusion flowchart

Figure 1 shows the patient selection flowchart. The treatment criteria were (1) aortic arch aneurysm diameter > 55 mm or an annual increase > 5 mm and (2) type non-A-non-B aortic dissection with three involved supra-arch branches. The exclusion criteria were (1) Stanford type A aortic dissection extending to the root of the ascending aorta, with insufficient normal anchoring area at the proximal end; (2) patients underwent open surgery and three-branch reconstruction using hybrid procedures, the chimney technique, or a physician-modified stent-graft (PMSG) technique; and (3) incomplete follow-up data. Thirty-six consecutive patients who underwent TEAAR via ISMNF using the triple (stiff)-sheathed gutter technique for aortic arch pathologies from August 2022 to April 2025 were included.

### Procedure

**Fig. 2:**
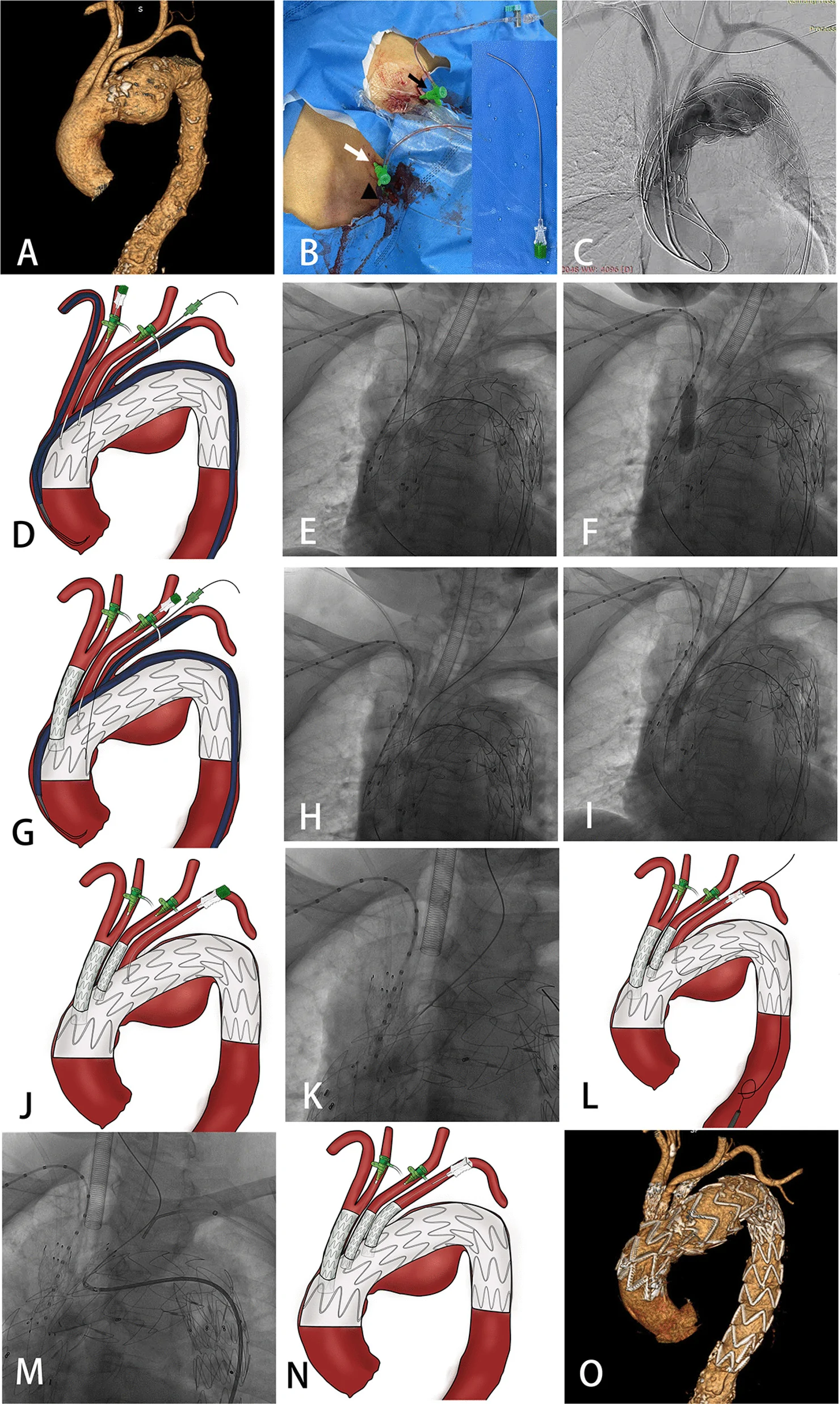
Surgical Illustration. **A** Pre-operative 3D reconstruction of the aortic arch. **B** After puncture of the right common carotid artery, the 6-Fr sheath was placed (black arrow). After puncture of left common carotid artery, the 6-Fr sheath was placed (white arrow). The left subclavian artery was punctured without removing the micro-puncture needle and 0.018-inch guide wire (white arrowhead). A pre-curved micro-puncture needle (black arrowhead) was introduced through the sheath. **C** DSA demonstrated patent cerebral blood flow through the gutter created by three 6-Fr, 90-cm-long sheaths without removing the dilator and Lunderquist wire (Cook Medical). **D** Illustration showing penetration of the right common carotid artery (RCCA). **E** A micro-puncture needle was introduced through the RCCA sheath. **F** Dilation with a 10-mm balloon. **G** Illustration showing penetration of the left common carotid artery (LCCA). **H** A micro-puncture needle was introduced through the LCCA sheath. **I** Dilation with an 8-mm balloon. **J** Illustration showing penetration of the left subclavian artery (LSA). **K** The trocar needle was introduced from the LSA access site over the previously placed 0.018-inch guidewire. **L** After puncture, a 300-cm guidewire was advanced into the aorta and captured via the femoral approach using a snare, establishing a through-and-through connection from the LSA to the femoral wire. **M** Deployment of the covered stent. **N** Illustration showing deployment of the LSA covered stent. **O** Postoperative 3D reconstruction of the aortic arch

All cases were discussed by a multidisciplinary board composed of vascular surgeons, interventional radiologists, cardiac surgeons, cardiologists, and anesthetists. All patients underwent routine preoperative evaluation and CTA (Fig. 2A). The procedures were performed by two interventional radiologists with over 20 years of experience in endovascular therapy. All procedures were performed under general anesthesia. Regional cerebral oxygen saturation (RCOS) was monitored continuously using the INVOS 5100c (Somanetics, Troy, MI, USA). Sensors were placed bilaterally on the forehead over the frontal cortex. Mean arterial pressure (MAP) in the bilateral common carotid arteries (CCAs) was monitored using the IPM12 Vet (Shenzhen Mindray Bio-Medical Electronics Co., Ltd., Guangdong, People’s Republic of China) with invasive arterial pressure transducers connected to intraoperative carotid arterial sheaths.

### Access preparation, intraoperative RCOS and MAP monitoring, and triple (stiff)-sheathed gutter technique

The bilateral CCAs, LSA, and right brachial artery were punctured to prepare for ISMNF. A 6-Fr sheath was inserted into each CCA to the aortic arch under fluoroscopic guidance (Fig. 2B). A 21-G, 7-cm micro-needle (Micropuncture Check-Flo Performer Introducer Set; Cook Medical, Bloomington, IN, USA) was used to puncture the LSA within the left supraclavicular fossa. After successful LSA puncture, a 0.018-inch guidewire was advanced into the ascending aorta. If implantation of a covered stent in the IA was necessary, the right brachial artery was exposed surgically.

To maintain cervical blood flow, three 6-Fr, 90-cm-long sheaths (Flexor® Check-Flo® Introducer; Cook Medical) were inserted into the ascending aorta, one in each radial artery and one in a femoral artery, without removing the dilator and Lunderquist wire (Cook Medical) (Fig. 2C). This approach enhanced the force exerted by the long sheath on the stent-graft and induced a gutter between the stent-graft and vessel wall. This technique was named “the triple (stiff)-sheathed gutter technique.”

### Main stent-graft implantation

Common femoral access for the aortic components was obtained using the percutaneous pre-closure technique (ProGlide; Abbott Vascular, Redwood City, CA, USA). A conformable stent-graft was passed into the ascending and descending aorta. The first stent-graft was deployed in the descending aorta and the second in the aortic arch, with the anchor area in the ascending aorta. The percentage of oversizing of the aortic stent-graft was 10–20%. For aortic arch aneurysm and lesions with a short or angulated landing zone, proximal oversizing was up to 20%.

### ISMNF of the IA, LCCA, and LSA

A 6-Fr sheath (TERUMO; Japan) was inserted from the right CCA access site into the IA over a guidewire to the level of the IA orifice. A pre-curved micro-puncture needle (21-G, 15-cm trocar needle, Skater Introducer Set; Argon Medical Devices, Inc., Frisco, TX, USA) was introduced through the sheath and rotated to determine the optimal puncture site (Fig. 2D and E). After successful stent-graft puncture, a 300-cm guidewire (V-18 Control Wire; Boston Scientific, Natick, MA, USA) was advanced into the aorta. The endograft fenestration was then sequentially predilated using 4–10-mm balloons (Fig. 2F), followed by bare metal stent deployment (E-Luminexx; Bard Peripheral Vascular, Tempe, AZ, USA) through the CCA approach or deployment of a 12–14-mm self-expandable covered stent (Fluency Plus, Bard Peripheral Vascular or Viabahn, WL Gore & Associates, Flagstaff, AZ, USA) through the right brachial approach. For each fenestrated supra-aortic branch, a covered stent was the first-choice bridging device.

A similar procedure was used for LCCA revascularization (Fig. 2G and H). However, the procedure differed from that used for the IA in that the covered stent (LifeStream™ Balloon Expandable Vascular Covered Stent; BD, Franklin Lakes, NJ, USA) was implanted through the 6-Fr sheath for left CCA stenting (Fig. 2I). According to the manufacturer’s instructions for use, a 5–8-mm covered stent may be post-dilated with balloons up to 10 mm in diameter. Following stent placement, the three 90-cm-long sheaths used to create the gutter were removed.

The stent-graft introducer was exchanged for a 20-Fr sheath (Braidin Pro; APT Medical, Shenzhen, China). A 21-G, 15-cm trocar needle (Skater Introducer Set) was used for LSA fenestration. The trailing end of this trocar needle was manually pre-curved based on the angle and direction between the LSA and aortic arch. The outer cannula of the trocar needle was introduced from the LSA access site over the previously placed 0.018-inch guidewire until its tip reached the aortic stent-graft. The tip was adjusted as perpendicular as possible to the aortic stent-graft through the tangential projection and axial view. After the 0.018-inch guidewire was removed, the needle core was introduced to penetrate the stent-graft (Fig. 2J and K). After puncture, a 300-cm guidewire (V-18 Control Wire; Boston Scientific) was advanced into the aorta. The guidewire was captured via the femoral approach using a snare (Atrieve Vascular Snare Kit; Argon Medical Devices, Inc.), establishing a through-and-through connection from the LSA to the femoral wire (Fig. 2L). The fenestrated endograft was sequentially predilated using 4–6-mm balloons, followed by deployment of a 10–12-mm self-expandable covered stent (Fluency Plus; Bard Peripheral Vascular) through the femoral 20-Fr sheath (Fig. 2M and N).

The LSA guidewire and sheaths in the bilateral CCAs were removed, and hemostasis was achieved by manual compression. The femoral access was closed with the ProGlide vascular closure device (Abbott Vascular), and the radial artery access was compressed using a radial artery compression device (TR Band; Terumo Corporation, Somerset, NJ, USA). The incisions were closed using a standard technique.

### Study endpoints, follow-up protocol, and definitions

Operative details, technical success, clinical outcomes, and adverse events were recorded. All patients underwent CT angiography at 3 and 12 months, and yearly thereafter (Fig. 2O). The patients received dual antiplatelet therapy (100 mg aspirin and 75 mg clopidogrel daily) for 6 months. Lifelong aspirin was continued thereafter. Intraoperatively, bilateral RCOS and MAP were monitored throughout the procedure. The times were recorded for interval 1 (from stent-graft deployment to the first balloon dilatation during IA fenestration) and interval 2 (from stent-graft deployment in the aortic arch to the first balloon dilatation during LCCA fenestration).

The primary outcomes were overall mortality, aorta-related mortality, and major adverse events (i.e., puncture site bleeding, stroke, spinal cord ischemia, retrograde type A aortic dissection [RTAD], and pneumothorax) at 30 days and during follow-up. The secondary endpoints were endoleaks, freedom from reintervention (time to event), and branch stent patency [14, 15]. Technical success was defined as successful completion of the procedure, with patent branch stents, no intraoperative deaths, no type I or III endoleaks observed on the final angiogram, and no conversion to surgical repair. Death within 24 h and type I or III endoleak were recorded in accordance with TEVAR reporting standards [14]. Aorta-related death was defined as death associated with thoracic aortic rupture, dissection, or infection. Branch stent assessment was based on CTA according to (1) < 50% in-stent stenosis on multiplanar reconstructions and (2) absence of stent fracture, collapse, or kinking.

### Statistical analysis

Continuous data are expressed as the mean ± standard deviation (range). Categorical data are presented as number (percentage). Comparisons of RCOS and MAP in the carotid arteries measured at different timepoints (before main stent-graft deployment [T1], after main stent-graft deployment [T2], and after in situ fenestration of the IA [T3], LCCA [T4], and LSA [T5]) were performed using repeated measures analysis of variance followed by the Bonferroni post hoc test. Kaplan–Meier analysis was used to estimate freedom from all-cause death and reintervention. Statistical analyses were performed using SPSS software, version 21.0 (IBM Corp., Armonk, NY, USA). All statistical tests were two-sided, and *p* < 0.05 was statistically significant.

## Results

### Patient demographics and procedural details

**Fig. 3:**
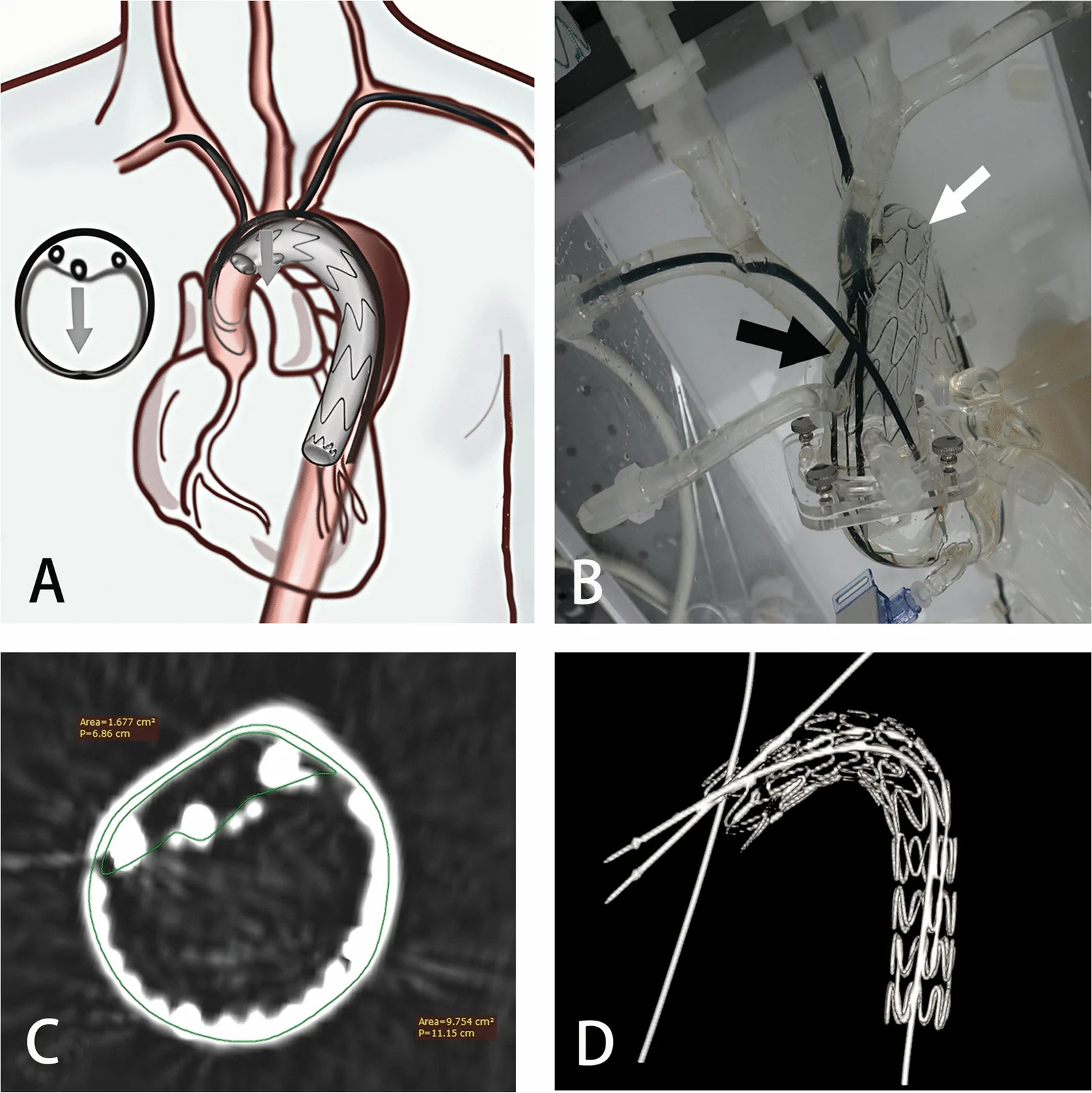
Computed tomography confirmed the gutter in an in vitro experiment. **A** Schematic diagram of gutter formation. The long sheaths exert a force (gray arrow) on the stent-graft. **B** Three 6-Fr long sheaths (black arrow) were inserted in the phantom of the aortic arch. **C**, **D** 3D reconstruction of the phantom. The ratio of its area to the corresponding aortic cross-sectional area was measured (17.2%, 1.677/9.754 cm^2^; gutter area: 1.462–1.685 cm.^2^)

The patients’ demographic characteristics are summarized in Table 1. Cervical branch revascularization was performed successfully in all cases. The technical success rate was 94.4% (34/36; 95% CI, 81.3–99.3%). Immediate type Ia endoleaks were identified on final angiography in two patients (2/36; 95% CI, 0.7–19.1%). These endoleaks required no intervention and resolved spontaneously during follow-up. The procedural details and devices are listed in Table 2. In an in vitro experiment (Fig. 3A and B), the gutter was confirmed by CT (Fig. 3C and D) and the ratio of its area to the corresponding aortic cross-sectional area was measured (17.2%, 1.677/9.754 cm^2^). The stent-graft (Lifetech Scientific Co., Ltd., 40–38 × 200 mm) was used.

**Table 1:** Patients’ demographic characteristics (*n* = 36)

Characteristic	Patients (*n* = 36)
Age (y)^*^	64.6 ± 12.53 (35, 85)
Sex	
Male	30 (83.3)
Female	6 (16.7)
Comorbidities	
Hypertension	24 (66.7)
Coronary artery disease	5 (13.9)
Diabetes mellitus	10 (27.8)
Hyperlipidemia	12 (33.3)
Stroke	5 (13.9)
COPD	10 (27.8)
Renal insufficiency	6 (16.7)
Diagnosis	
Aortic arch aneurysm	27 (75.0)
Non-A non-B AD	9 (25.0)
Arch type	
I	9 (25.0)
II	21 (58.3)
III	3 (16.7)

Note: Unless otherwise specified, data are presented as number (%). ^*^Data presented as mean ± standard deviation (range). *y* years, *COPD* chronic obstructive pulmonary disease, *AD* aortic dissection

**Table 2:** Details of the procedures and devices for 36 patients who underwent endovascular aortic arch repair in zone 0 (*n* = 36)

Detail	Patients (*n* = 36)
Aortic stent-graft	51 (100.0)
Valiant, Medtronic Vascular, Santa Rosa, CA, USA	36 (70.6)
Hercules	15 (29.4)
Device for the IA reconstruction	
Fluency (Bard Peripheral Vascular, Temple, AZ)	20 (55.5)
Viabahn (Gore, Flagstaff, AZ, USA)	6 (16.7)
E-Luminexx (Bard Peripheral Vascular, Temple, AZ)	10 (27.8)
Device for the LCCA reconstruction	
LifeStream™ (Bard Peripheral Vascular, Temple, AZ)	36 (100.0)
Device for the LSA reconstruction	
Fluency (Bard Peripheral Vascular, Temple, AZ)	28 (77.8)
Viabahn (Gore, Flagstaff, AZ, USA)	8 (22.2)
Operation time (minutes)^*^	218.4 ± 62.9 (148.0, 480.0)
Fluoroscopy time (minutes)^*^	56.4 ± 11.3 (37.0, 84.0)
Mean blood loss (mL)	177.8 ± 92.2 (90.0, 500.0)
Amount of contrast used (mL)	135.0 ± 14.6 (110.0, 170.0)
Interval 1 (minutes)	10.5 ± 2.7 (7.0, 19.0)
Interval 2 (minutes)	19.5 ± 3.1 (14.0, 26.0)

Note: Unless otherwise specified, data are presented as number (%). ^*^Data are presented as mean ± standard deviation (range). *IA* innominate artery, *LCCA* left common carotid artery, *LSA* left subclavian artery

**Fig. 4:**
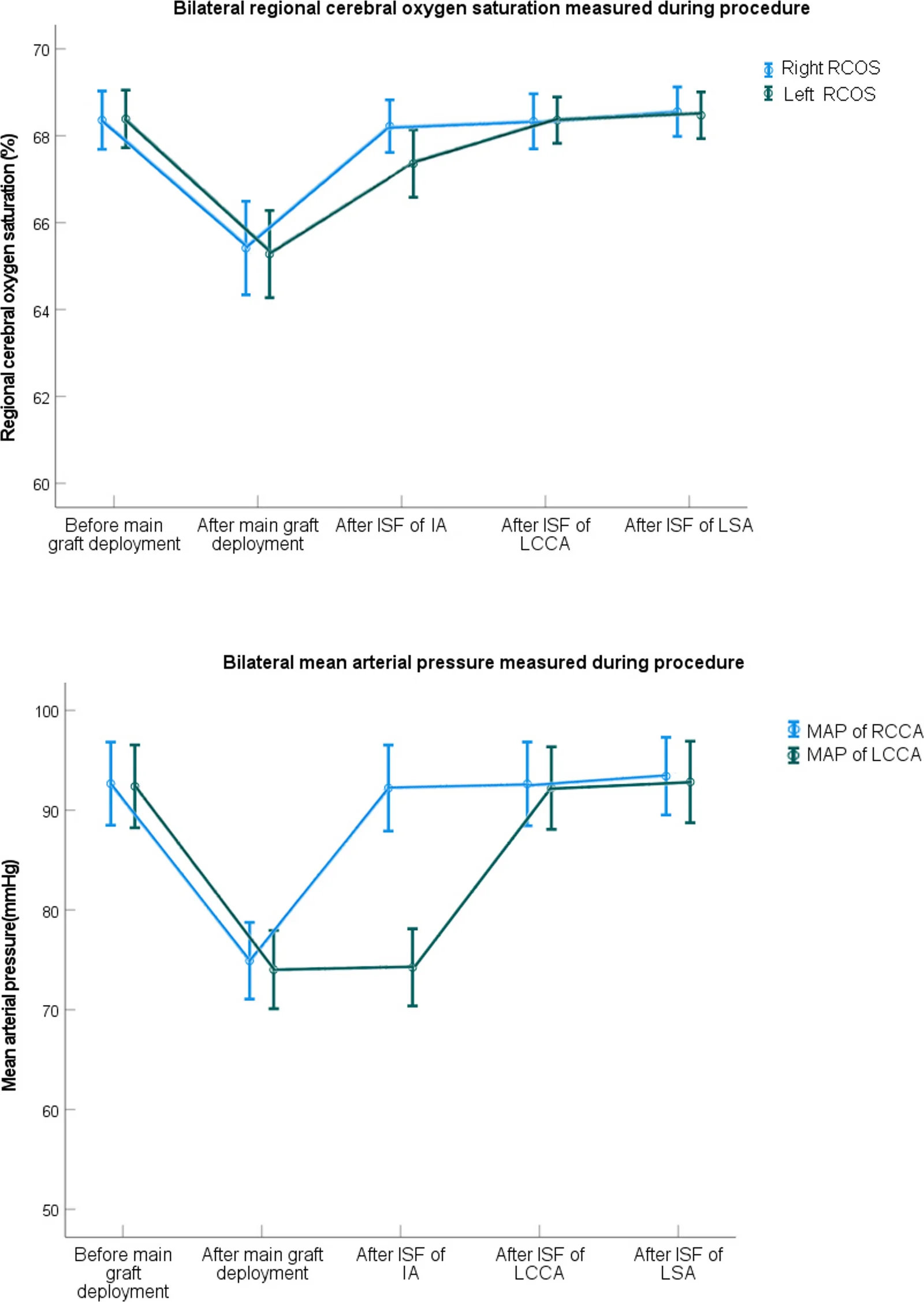
Continuous data for bilateral regional cerebral oxygen saturation (RCOS) and mean arterial pressure (MAP) in the bilateral common carotid artery measured during the procedure

Continuous data for RCOS and MAP (mmHg) were recorded for the bilateral CCAs (Fig. 4). Before versus after aortic stent-graft deployment, RCOS (right: 0.684 ± 0.015 vs. 0.654 ± 0.024, *p* < 0.05; left: 0.684 ± 0.015 vs. 0.653 ± 0.022, *p* < 0.05) and MAP (right: 92.67 ± 9.181 vs. 74.92 ± 8.470 mmHg, *p* < 0.05; left: 92.39 ± 9.141 vs. 74.03 ± 8.640 mmHg, *p* < 0.05) decreased in both CCAs. Before versus after IA fenestration, RCOS increased bilaterally (right: 0.654 ± 0.024 vs. 0.682 ± 0.013, *p* < 0.05; left: 0.653 ± 0.022 vs. 0.674 ± 0.017, *p* < 0.05). MAP in the right CCA increased (74.92 ± 8.470 vs. 92.22 ± 9.502 mmHg, before versus after reconstruction, respectively; *p* < 0.05). However, no significant change occurred on the left side (74.03 ± 8.640 vs. 74.25 ± 8.513 mmHg; *p* = 1.000). After LCCA revascularization, there was no change in RCOS and MAP in the right CCA (RCOS: 0.682 ± 0.013 vs. 0.683 ± 0.014, *p* = 0.422; MAP: 92.22 ± 9.502 vs. 92.64 ± 9.249 mmHg, *p* = 1.000), while RCOS and MAP in the LCCA increased (RCOS: 0.674 ± 0.017 vs. 0.684 ± 0.012, *p* < 0.05; MAP: 74.25 ± 8.513 vs. 92.22 ± 9.097 mmHg, *p* < 0.05). The mean ischemia time for the IA (interval 1) was 10.5 ± 2.7 min and that for the LCCA (interval 2) was 19.5 ± 3.1 min.

### 30-day outcomes

The 30-day and follow-up outcomes are shown in Table 3. The median follow-up period was 19 months (range, 1–32). One patient with aortic arch aneurysm died of severe pneumonia despite successful endovascular exclusion, leading to a 30-day mortality rate of 2.8% (1/36; 95% CI, 0.1–14.9%). The overall stroke rate was 8.3% (3/36; 95% CI, 1.8–22.5%). One patient presented with major stroke from middle cerebral artery branch occlusion, and patient underwent emergent mechanical thrombectomy and showed an improvement in the modified Rankin Scale from 4 at presentation to 2 at discharge. Two other patients experienced minor stroke caused by lacunar infarctions in the basal ganglia and fully recovered by the time of discharge. No cases of access-related adverse events, RTAD, or spinal cord ischemia were recorded.

**Table 3:** Follow-up details for 36 patients who underwent endovascular aortic arch repair in zone 0 (*n* = 36)

	*n* = 36
30-day outcomes	
Technical success	34 (94.4)
30-day mortality	1 (2.8)
Immediate type Ia endoleak	2 (5.6)
Puncture site complications	0 (0.0)
Bleeding/pseudoaneurysm	0 (0.0)
Pneumothorax	0 (0.0)
Postoperative major adverse events	
Stroke	3 (8.3)
RTAD	1 (2.8)
Spinal cord ischemia	0 (0.0)
Midterm outcomes	
Follow-up (months)^*^	18.2 ± 8.7 (1, 32)
All cause death	4 (11.1)
Aorta-related death	0 (0.0)
Branched stent/graft occlusion	0 (0.0)
Branched stent/graft fracture	0 (0.0)
Follow-up endoleak	
Type Ia	2 (5.6)
Type Ib	3 (8.3)
Type Ic	0
Type II	0
Major adverse events	
Stroke	3 (8.3)
RTAD	1 (2.8)
Spinal cord ischemia	0 (0.0)
Reintervention	5 (13.9)

Note: Unless otherwise specified, data are presented as number (%). ^*^Data are presented as mean ± standard deviation (range). *RTAD* retrograde type A aortic dissection

### Midterm outcomes

The all-cause death rate was 11.1% (3/36; 95% CI, 1.8–22.5%). Three additional deaths occurred during follow-up. One patient died of cerebral hemorrhage at 18 months, one died of liver carcinoma at 24 months, and one died of congestive heart failure at 32 months. In addition to two immediate type Ia endoleaks, three cases of type Ib endoleaks were detected on follow-up CT. These three patients were diagnosed with aortic dissection and underwent TEVAR at the distal end of the primary stent-graft. No other cases of endoleak were identified. One patient experienced RTAD during the 3-month follow-up period and subsequently underwent successful open surgical repair. The total reintervention rate was 13.9% (5/36; 95% CI, 4.7–30.4%). Apart from the one patient who underwent emergency thrombectomy for major stroke and one with RTAD, all reinterventions were performed for distal disease progression. No branch stent stenosis, obstruction, or fracture occurred.

**Fig. 5:**
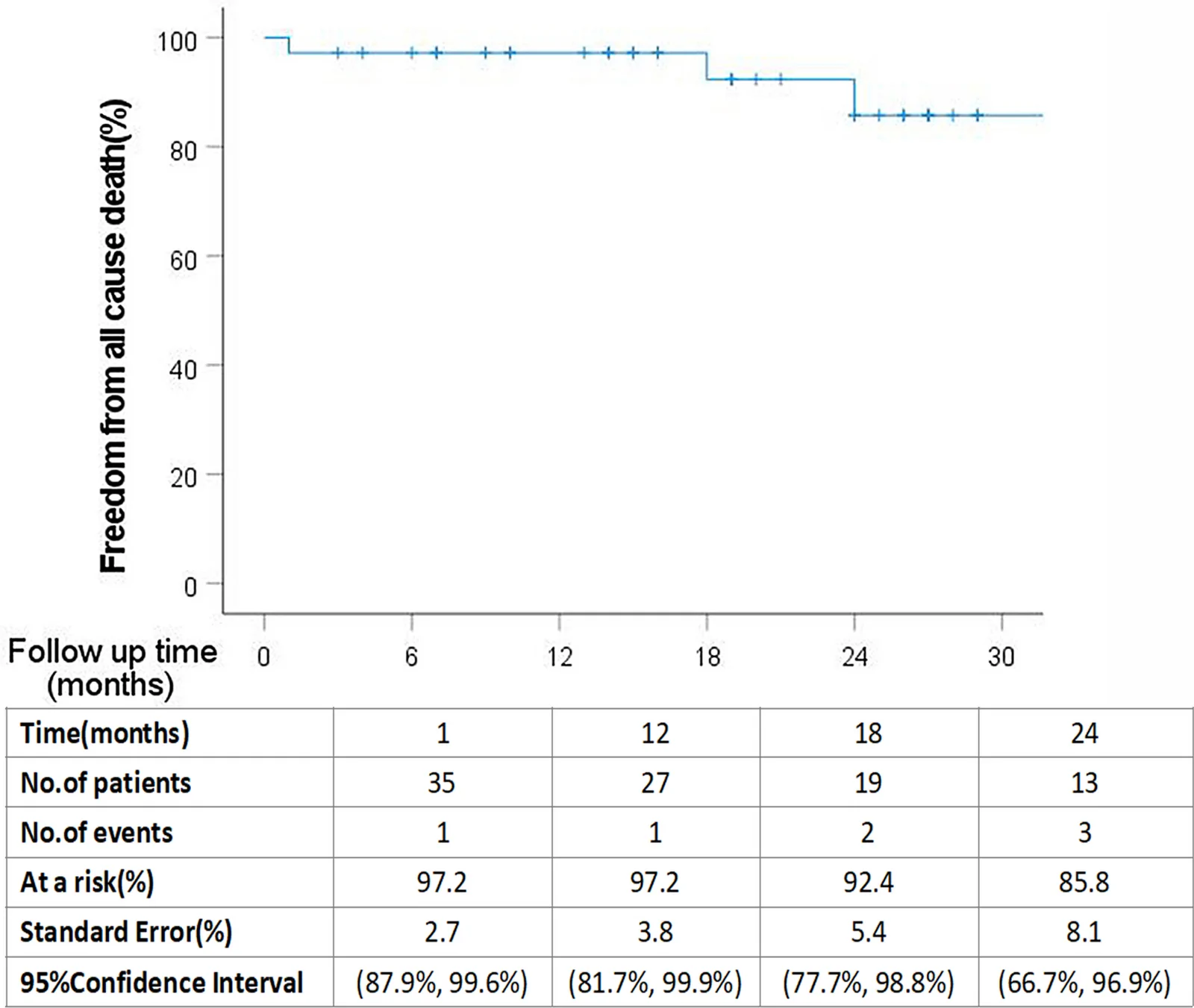
Kaplan–Meier survival curve for all-cause death

**Fig. 6:**
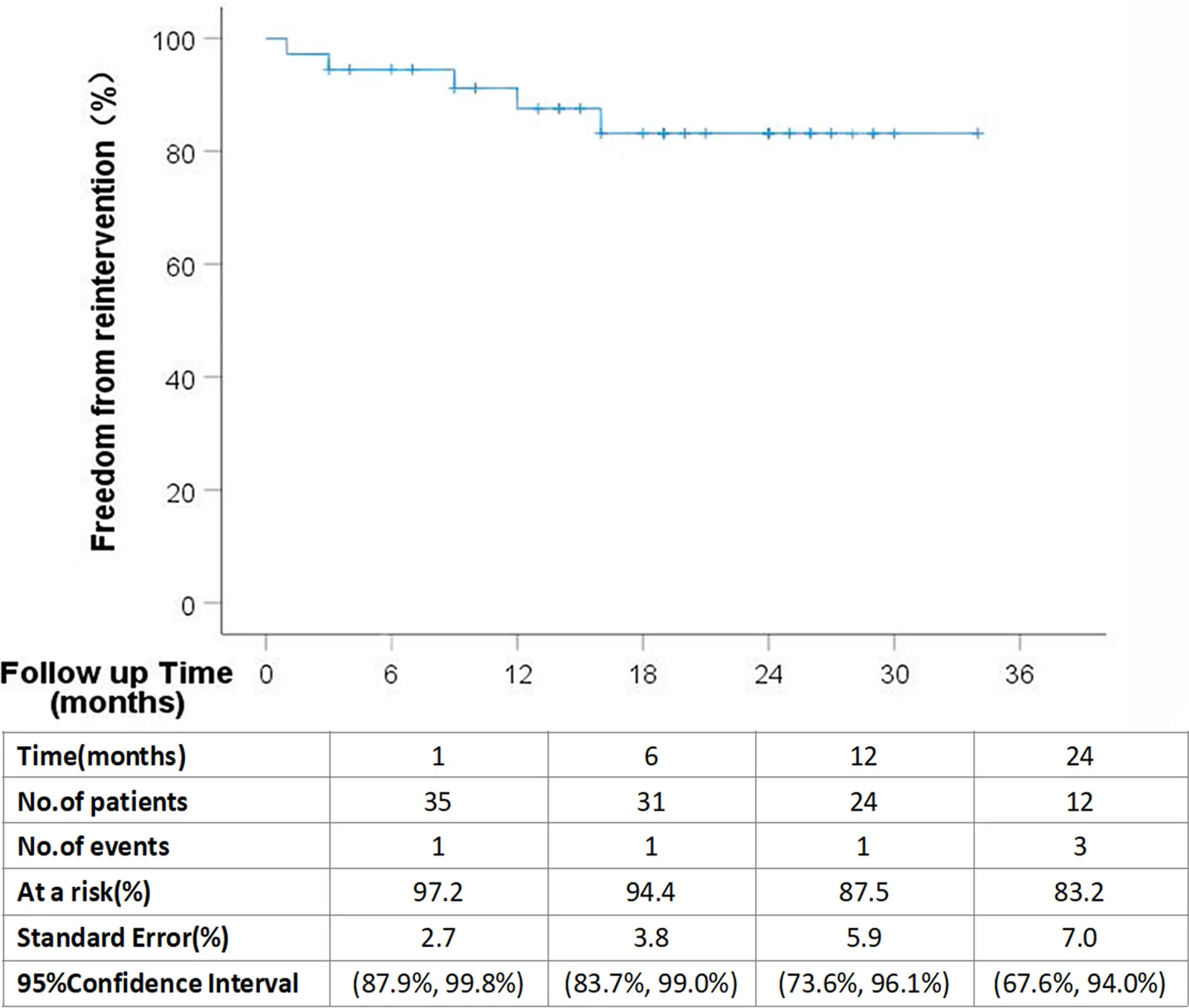
Kaplan–Meier survival curve for reintervention

Figure 5 shows the Kaplan–Meier survival curve for all-cause death. The 1- and 2-year survival rates were 97.3% ± 2.7% and 85.8% ± 8.1%, respectively. Figure 6 shows the Kaplan–Meier survival curve for reinterventions. The 1- and 2-year cumulative rates of freedom from reintervention were 87.5% ± 5.9% and 83.2% ± 7.0%, respectively.

## Discussion

As a minimally invasive procedure, TEAAR has become increasingly important for the treatment of aortic pathologies. Both energy-based techniques (i.e., radiofrequency, laser energy) and sharp techniques (i.e., needles, wires) have been used to create fenestrations in the fabric of the stent-graft. Previous studies on TEAAR with in situ needle fenestration have reported satisfactory results, with success rates of 85.7–100.0%, adverse event rates of 13.3–28.6%, reintervention rate of 0.0–4.2%, and 1-year survival rates of 92.3–94.6% [4–6, 8–18]. This study reported acceptable outcomes, which are comparable to those reported previously.

Puncture devices play a crucial role in in situ needle fenestration. Previous studies have used different puncture systems for cervical branch revascularization compared with those used in this study. The reported puncture systems for the IA or left CCA include percutaneous transhepatic cholangiography needles, aspiration needles, and liver biopsy needles [4–6, 8, 9]. Previous studies have reported LSA fenestrations performed via the brachial artery approach [5, 6, 8, 19]. In the present study, a single type of micro-puncture needle was used to revascularize all cervical branches. 21-G trocar needle can be pre-curved prior to introduction to adapt to the angle between the IA (LCCA, or LSA) and aortic arch. The route from the LSA puncture site (left supraclavicular fossa) to the endograft penetration site was short, resulted in a longitudinal mechanical advantage for puncture.

Stroke remains a concerning adverse event after TEVAR, with published rates of 9–28.6% [1, 2, 4–9, 11–13]. Potential causes of stroke include intraoperative embolization (especially atherosclerotic plaque emboli), insufficient cerebral perfusion, and thrombogenesis owing to manipulation of the supra-aortic vessels. Stroke rates differ depending on the technique used. The reported incidence of stroke with hybrid endovascular repair for aortic arch pathology ranges from 5.7 to 14.3% [19, 20]. Studies have reported a stroke rate of 3.7–6.0% [11, 12, 21, 22] for PMSG and 10.5% [23] for inner branched endoprosthesis for the treatment of aortic arch pathologies. In the present study, the overall incidence of stroke was higher than that observed with PMSG. The possible reasons are as follows. First, TEVAR performed using PMSG does not involve cerebral ischemia time. Conversely, in situ fenestration during endovascular manipulation entails more handling of supra-aortic vessels than PMSG, thereby increasing the risk of cerebral infarction owing to embolus detachment. One patient in the present study with an atherosclerotic aortic arch experienced a major stroke during the procedure. It was assumed that excessive manipulation of the puncture needle, catheters, and guidewires within the atherosclerotic arch may have contributed to the thrombosis, resulting in stroke.

A critical consideration during ISMNF is the maintenance of sufficient cerebral perfusion. Various methods to maintain cerebral blood flow have been reported, including establishment of extracorporeal circulation or external flow (surgical bypass) and endovascular shunting using physician-modified devices (sheaths and external tubes) [5, 7, 24, 25]. Another method to avoid cerebral blood flow occlusion is gutter creation between the stent-graft and vessel wall. A previous study reported the gutter balloon technique for brain protection [9]. An advantage of the balloon gutter is that less manipulation with stiff wires in the supra-aortic branches and aortic arch is required, which might be associated with a lower risk of plaque disruption and embolic stroke than the triple-sheathed technique. However, the balloon itself occupies space in the gutter, which may result in partial occlusion of cerebral blood flow. In this study, three long sheaths, without removal of the dilator, and the stiff guidewire provided sufficient force on the stent-graft to create a gutter between the stent-graft and vessel wall.

The RCOS and MAP of bilateral CCAs were monitored continuously throughout ISMNF. A decrease in RCOS of < 20% from baseline has negative predictive value for intraoperative neurological adverse events [26, 27]. In the present technique, bilateral CCA RCOS decreased slightly after coverage of the aortic arch. These findings indicate the beneficial effect of the gutter. In the RCOS and MAP measurement curves, cerebral blood flow was more directly reflected by MAP than RCOS. After IA revascularization, although blood flow was restored only in the IA, CCA RCOS increased bilaterally because of the presence of intracranial anterior circulation.

The present study had several limitations. First, there are some disadvantages of this technique. Extensive manipulation with needles, guidewires, and sheaths in the supra-aortic branches and the aortic arch is associated with risks of plaque disruption and embolic stroke, especially in patients with shaggy aortas. Placing multiple long sheaths and super-stiff guidewires in the ascending aorta carries a potential risk of injuring the ascending aorta and causing new-onset dissection. Second, in patients with type III aortic arch, the larger angles between the branch vessels and the aorta may increase the risk of puncture failure.

## Conclusions

In conclusion, TEAAR via ISMNF with the triple (stiff)-sheathed gutter technique was performed successfully in aortic arch pathologies, with acceptable early and midterm outcomes, and it maintained adequate cerebral blood flow intraoperatively.

## Data Availability

The datasets generated and/or analyzed during the current study are not publicly available due to ethical restrictions and patient confidentiality requirements but are available from the corresponding author on reasonable request.
